# Clinical Outcomes and Factors Associated with Response to Fluoroscopy-Guided Cervical Medial Branch Pulsed Radiofrequency in Chronic Cervical Facet Joint Pain: A Retrospective Cohort Study

**DOI:** 10.3390/medicina62091770

**Published:** 2026-09-15

**Authors:** Nevcihan Şahutoğlu Bal, Şükriye Dadalı, Ali Çoştu, Gülçin Babaoğlu, Ülkü Sabuncu, Emel Başar, Erkan Yavuz Akçaboy

**Affiliations:** Department of Pain Medicine, Ankara Bilkent City Hospital, 06800 Ankara, Türkiye; sukriyedadali@gmail.com (Ş.D.); alicostu@gmail.com (A.Ç.); gulcinpektasli@gmail.com (G.B.); sabuncuulku@gmail.com (Ü.S.); dr_emel86@hotmail.com (E.B.); akcaboyyavuz@gmail.com (E.Y.A.)

**Keywords:** cervical facet joint pain, cervical medial branch, pulsed radiofrequency, chronic neck pain, fibromyalgia, meaningful pain relief, Neck Disability Index

## Abstract

*Background and Objectives*: Pulsed radiofrequency (PRF) of the cervical medial branches is a non-destructive neuromodulatory option for chronic cervical facet joint pain, yet evidence regarding the durability of clinical response and factors associated with sustained benefit remains limited. This study aimed to characterize the 6-month trajectory of pain and disability after fluoroscopy-guided cervical medial branch PRF and to explore baseline factors associated with meaningful pain relief (MPR). *Materials and Methods*: In this retrospective single-center cohort study, 75 patients with chronic cervical facet joint pain who achieved ≥50% temporary pain relief following a diagnostic medial branch block underwent fluoroscopy-guided cervical medial branch PRF, followed by injection of local anesthetic and corticosteroid at the treated levels. Numeric Rating Scale (NRS) and Neck Disability Index (NDI) scores were evaluated at baseline and at 1, 3, and 6 months. MPR was defined as ≥50% reduction in NRS from baseline. Global Perceived Effect (GPE) and changes in analgesic medication use were assessed as complementary clinical outcomes. An exploratory multivariable logistic regression analysis was performed to examine factors independently associated with MPR at 6 months. *Results*: Median NRS scores decreased from 7.0 at baseline to 4.0, 4.0, and 5.0 at 1, 3, and 6 months, respectively, while median NDI scores decreased from 30.0 to 14.0, 16.0, and 20.0 (both overall *p* < 0.001). Despite sustained improvement relative to baseline, MPR progressively declined from 57.3% at 1 month to 49.3% at 3 months and 36.0% at 6 months (overall *p* < 0.001). Favorable GPE and reduced analgesic medication use showed parallel declines, supporting attenuation of overall clinical benefit over time. In the exploratory multivariable model, fibromyalgia was independently associated with 75% lower odds of 6-month MPR (OR = 0.25, 95% CI: 0.088–0.709; *p* = 0.009). Age, sex, and baseline NRS were not significantly associated with 6-month MPR. No serious acute periprocedural adverse events were documented during the 2 h observation period. *Conclusions*: Fluoroscopy-guided cervical medial branch PRF, combined with post-procedural corticosteroid and local anesthetic administration, was associated with clinically meaningful improvements in both pain and neck-related disability, with the greatest benefit during the first 3 months and partial attenuation by 6 months. The progressive decline across pain response, patient-reported global improvement, and analgesic-use outcomes highlights the time-dependent nature of the benefit associated with the combined intervention. Fibromyalgia was independently associated with substantially lower odds of 6-month MPR in this exploratory analysis; this association should be interpreted cautiously and requires prospective validation before it can inform patient selection, although it may be relevant when counseling patients regarding the durability of response.

## 1. Introduction

Chronic neck pain remains one of the most prevalent musculoskeletal conditions worldwide, and its burden continues to grow: global estimates indicate that 203 million people were affected in 2020, an increase of 77.3% since 1990, with projections suggesting a further rise to 269 million cases by 2050 [1]. The condition disproportionately affects women and peaks during the working years, between 45 and 74 years of age, underscoring its considerable socioeconomic impact [1].

Among the numerous structures capable of generating chronic axial and referred neck pain, the cervical facet (zygapophysial) joints are recognized as a common source, innervated by the medial branches of the cervical dorsal rami. Current evidence-based guidelines support diagnostic cervical medial branch blocks as a reliable means of confirming facet joint pain, with Level II evidence and moderate strength of recommendation, and similarly support cervical radiofrequency ablation as a therapeutic option once the diagnosis has been established, again with Level II evidence [2,3].

When conservative treatments—oral medication, physical therapy, and facet joint injections—fail to provide durable relief, and diagnostic medial branch blocks confirm a facet joint origin, interventional neuromodulation becomes the next reasonable step. Among the available radiofrequency techniques, pulsed radiofrequency (PRF) has emerged as a minimally invasive alternative to conventional (thermal) radiofrequency ablation. Rather than relying on continuous heat to coagulate neural tissue, PRF delivers brief electrical pulses interspersed with resting phases, exposing the target nerve to an electric field while limiting the temperature rise associated with irreversible thermal injury [4].

The mechanisms underlying PRF’s analgesic effect are still being clarified, but converging evidence points to a predominantly neuromodulatory, rather than ablative, action. Experimental work has demonstrated that PRF exposure induces measurable ultrastructural changes within axons—including mitochondrial membrane abnormalities and disorganization of microfilaments and microtubules—that are more pronounced in C-fibers than in A-delta or A-beta fibers, suggesting a degree of selectivity for nociceptive pathways [5]. Complementary work in animal models has shown that both pulsed and continuous radiofrequency current applied adjacent to the cervical dorsal root ganglion induce delayed cellular activity within the dorsal horn, indicating that radiofrequency-induced neuromodulatory effects can persist for several days after exposure regardless of the radiofrequency modality used [6].

A recent systematic review and meta-analysis of radiofrequency neurotomy for chronic neck pain rated the overall evidence as Level II with moderate strength of recommendation, based on a limited number of randomized and observational studies, and highlighted a persistent paucity and heterogeneity of the available literature [7]. Within this body of evidence, studies specifically addressing cervical medial branch PRF remain comparatively scarce and heterogeneous in design. Yen et al. evaluated 204 patients with cervical facet joint pain, including 157 who underwent medial branch block followed by PRF and identified sagittal alignment parameters—including cervical lordosis and the C2–C7 Cobb angle—as independent predictors of outcome [8], while Chang and Yang, in a smaller retrospective cohort of 21 patients, reported a significant reduction in pain scores at 1 and 3 months, with 52.4% of patients achieving clinically meaningful relief at 3 months [9]. More recently, a retrospective cohort study using a comparable follow-up schedule and the same definition of meaningful pain relief (≥50% reduction in pain at 6 months) to the present study reported a 6-month success rate of 35.2% and identified paraspinal tenderness, shorter pain duration, and lower baseline pain and disability as independent predictors of treatment success [10].

Despite this growing body of evidence, most existing studies have focused on structural, procedural, or symptom-duration-related predictors of PRF outcome, while comorbidity-based predictors—including conditions associated with central pain amplification—have received comparatively little attention. Moreover, few studies have evaluated pain intensity, functional disability, and patients’ global perceived effect together over a 6-month horizon within the same cohort. The primary aim of this study was therefore to evaluate changes in pain intensity following cervical medial branch PRF combined with post-procedural local anesthetic and corticosteroid administration in patients with chronic cervical facet joint pain. Secondary aims were to assess changes in functional status, global perceived effect, and analgesic medication use at 1, 3, and 6 months, and to explore baseline clinical and demographic factors—including comorbidities—associated with achieving meaningful pain relief at 6 months.

## 2. Materials and Methods

### 2.1. Study Design and Setting

This retrospective cohort study was conducted at the Department of Pain Medicine, Ankara Bilkent City Hospital, Ankara, Türkiye. Data were extracted from electronic hospital records for patients who underwent cervical medial branch PRF for chronic cervical facet joint pain between 2020 and 2025. The study was conducted in accordance with the Declaration of Helsinki and was approved by the Institutional Review Board of Ankara Bilkent City Hospital (protocol code TABED 2-26-1868, approved on 21 January 2026).

### 2.2. Participants

Of the 103 patients who underwent fluoroscopy-guided cervical medial branch PRF for chronic cervical facet joint pain at the study institution between 2020 and 2025, 75 met all inclusion criteria and were included in the final analysis. Thirteen patients were excluded because of missing 6-month follow-up data; five patients were excluded because they met one or more of the predefined exclusion criteria described below, most commonly radicular pain attributable to disc herniation or active inflammatory rheumatologic disease requiring systemic corticosteroid treatment; and ten patients were excluded because of receipt of another invasive cervical procedure in close temporal proximity to PRF (cervical epidural steroid injection, *n* = 3; trigger point injections, *n* = 7). The participant flow is summarized in Supplementary Figure S1.

Patients were included if they met all of the following criteria: age ≥18 years; a clinical diagnosis of cervical facet joint pain, corroborated by magnetic resonance imaging (MRI) findings of facet joint degeneration; chronic neck pain of at least 3 months’ duration; an axial neck pain pattern and/or pain referred from the neck to the head consistent with a facet origin; a reported ≥50% temporary pain reduction after a diagnostic cervical medial branch block; an inadequate response to conservative treatment; PRF performed at the study institution between 2020 and 2025; and complete pre- and post-procedural Numeric Rating Scale (NRS) data at 1, 3, and 6 months, together with Neck Disability Index (NDI), Global Perceived Effect (GPE), and Likert-scale medication-use data and complete procedural records of the PRF parameters applied.

All patients underwent a diagnostic cervical medial branch block prior to PRF, performed by pain specialists using the same fluoroscopy-guided technique subsequently used for the PRF procedure, with infiltration of 0.5–1 mL of 1% lidocaine, prepared by dilution of 2% lidocaine hydrochloride (Aritmal^®^ 2% lidocaine hydrochloride, Osel İlaç San. ve Tic. A.Ş., İstanbul, Turkey) at the target level(s). Only patients who reported ≥50% temporary pain relief following this diagnostic block were considered for PRF.

Patients were excluded if they had a history of previous cervical spine surgery (discectomy, fusion, laminectomy, or instrumentation); radicular pain attributable to cervical disc herniation, foraminal stenosis, or central canal stenosis; a secondary cause of neck pain attributable to infection, tumor, active inflammatory rheumatologic involvement of the cervical spine, or trauma; active infection, coagulopathy, or inability to discontinue anticoagulant therapy; severe psychiatric illness or poor follow-up compliance; missing 6-month follow-up data; incomplete technical records of the PRF procedure; or receipt of another invasive cervical procedure (e.g., epidural steroid injection, trigger point injection, or facet joint injection), other than the diagnostic medial branch block, in close temporal proximity to PRF.

Patients with bilateral cervical facet joint pain were not excluded from the study. In these patients, the side with more prominent clinical findings, based on symptom severity, imaging findings, and/or palpation tenderness, was selected as the index treated side for the present analysis. If the contralateral side was treated at a separate session when clinically indicated, that subsequent procedure was not included as an additional observation. Accordingly, each of the 75 patients contributed only one treated side and one set of follow-up outcomes to the analysis.

Fibromyalgia and rheumatoid arthritis diagnoses were identified from pre-existing ICD-10 diagnoses established by the relevant specialty clinics (Rheumatology and/or Physical Medicine and Rehabilitation) within the same institution, as most patients in this cohort were co-managed across these departments. For fibromyalgia, the diagnosis was further corroborated by the treating pain physician’s own clinical findings of widespread pain and tenderness, documented in the procedural notes. For rheumatoid arthritis, all patients had disease that was not in an active inflammatory phase and did not involve the cervical spine at the time of PRF, consistent with the study’s exclusion criteria.

Only patients with complete outcome data at all follow-up time points were included in the analysis. No a priori sample size calculation was performed; the study included all eligible patients treated during the study period.

### 2.3. Procedure

All cervical medial branch PRF procedures were performed by pain specialists at our clinic using a standardized institutional protocol under fluoroscopic guidance. Patients were positioned supine, with the head supported in a neutral position. The skin overlying the target levels was prepared under aseptic conditions using 10% povidone–iodine solution.

**Figure 1:**
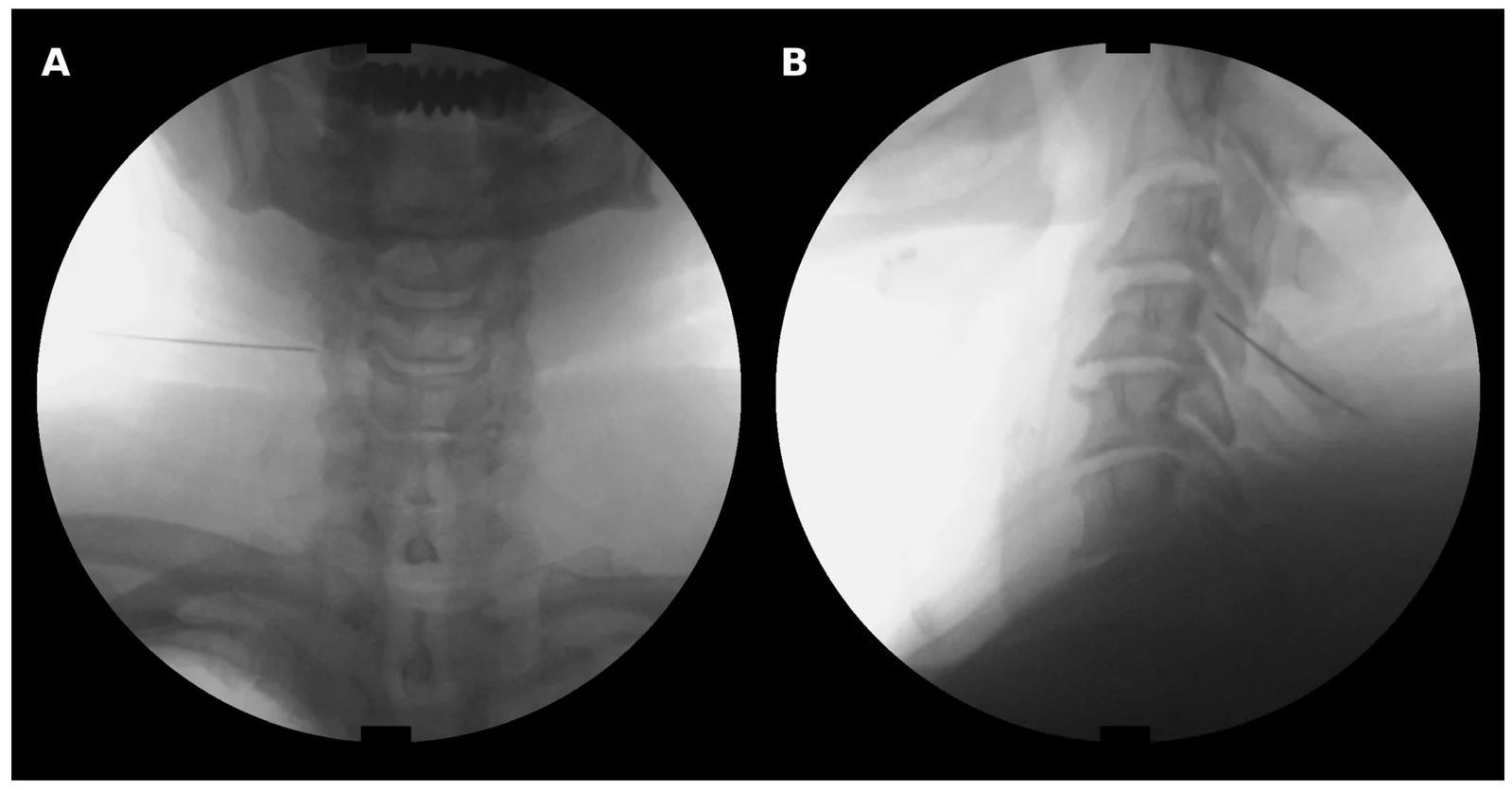
Representative fluoroscopic images of cervical medial branch pulsed radiofrequency. (**A**) Anteroposterior and (**B**) Lateral/oblique fluoroscopic views demonstrating cannula placement at the targeted cervical medial branches.

Fluoroscopic imaging was initially optimized by adjusting the C-arm angulation to align the superior and inferior vertebral endplates at the target levels. A lateral–oblique projection was then obtained to optimize visualization of the cervical facet joints and guide the cannula trajectory toward the corresponding medial branch target. Three contiguous cervical facet joint levels were targeted per patient—most commonly C3–4, C4–5, and C5–6 (*n* = 44, 58.7%) or, in patients with clinical or diagnostic-block findings extending to the cervicothoracic junction, C4–5, C5–6, and C6–7 (*n* = 31, 41.3%). Following skin and subcutaneous infiltration with 1% lidocaine (diluted from Aritmal^®^ 2%, Osel İlaç San. ve Tic. A.Ş., İstanbul, Turkey), a 22-gauge radiofrequency cannula (5 or 10 cm length, 5 mm active tip) was advanced under fluoroscopic guidance toward each target medial branch until appropriate bony contact was obtained. Final cannula position relative to the corresponding articular pillar was then verified using the previously optimized anteroposterior (AP) fluoroscopic projection, consistent with previously described fluoroscopy-guided cervical medial branch techniques [11]. Representative fluoroscopic images demonstrating cannula positioning at the targeted cervical medial branches are shown in Figure 1.

Following fluoroscopic confirmation of cannula position, cannula impedance was confirmed to be below 500 Ω before proceeding to sensory stimulation. Correct cannula placement was further verified using sensory stimulation (50 Hz, 0.3–0.5 V), with concordant paresthesia reproduced in the corresponding cervical region, followed by motor stimulation (2 Hz, 1.5–2.0 V) to confirm the absence of unintended motor responses in the upper-extremity musculature.

PRF was applied using a standard radiofrequency generator (NT1100™, Abbott Medical, Plymouth, MN, USA) for 360 s per level (pulse frequency, 2 Hz; pulse width, 20 ms; maximum output voltage, 45 V; electrode-tip temperature ≤ 42 °C). Following completion of PRF, a 3 mL mixture consisting of 1 mL of 2% lidocaine hydrochloride (Aritmal^®^) and 2 mL of dexamethasone (8 mg in total) was prepared in a single syringe for procedural convenience and sterility. Approximately 0.5 mL of this mixture was administered at each treated level through the RF cannula immediately before cannula removal. Any unused portion of the prepared mixture was discarded in accordance with standard sterile technique. At each treated level, PRF was applied to the medial branches of the spinal nerves immediately superior and inferior to the target facet joint (e.g., the C3–4 facet joint was treated via the medial branches of the C3 and C4 dorsal rami), consistent with standard cervical medial branch technique. Each procedure targeted a single side per treatment session. In patients with bilateral symptoms, treatment was performed unilaterally; any subsequent contralateral procedure, when clinically indicated, was performed at a separate session and was not included as an additional observation in the present analysis. All patients were monitored for acute periprocedural adverse events for 2 h following the procedure, and any events occurring during this period were recorded from the procedural notes. Transient early post-procedural pain flare, typically resolving within several days, was noted in the clinical records of a subset of patients but was not systematically captured beyond this acute observation window.

### 2.4. Outcome Measures

Pain intensity was assessed with the NRS (0–10; 0 = no pain, 10 = worst imaginable pain) at baseline and at 1, 3, and 6 months [12]. Functional status was assessed with the NDI (10 items, each scored 0–5; total range 0–50, with higher scores indicating greater disability), categorized as no disability (0–4), mild disability (5–14), moderate disability (15–24), severe disability (25–34), or complete disability (≥35) [13]. A 7-point Likert-type scale was used to record GPE, with percentage-based anchors as follows: 7 = very much improved (≥75% improvement); 6 = much improved (50–74% improvement); 5 = slightly improved (25–49% improvement); 4 = no change (0–24% change); 3 = slightly worsened (25–49% worsening); 2 = much worsened (50–74% worsening); 1 = very much worsened (≥75% worsening) [14]. Change in analgesic medication use was recorded using a 3-point Likert-type scale at 1, 3, and 6 months (0 = medication discontinued, 1 = medication use decreased, 2 = no change in medication use). Meaningful pain relief (MPR) was defined a priori as a ≥50% reduction in NRS score relative to baseline, assessed at 1, 3, and 6 months. No patients reported worsening on the GPE scale (categories 1–3) or increased analgesic medication use at the 1-, 3-, or 6-month follow-up assessments; these categories, although part of the original scales, were not observed in this cohort. Follow-up assessments (NRS, NDI, GPE, and analgesic medication use) were recorded in person during scheduled outpatient pain clinic visits at 1, 3, and 6 months by pain specialists within the treating department, who were not necessarily the same physician who had performed the index procedure.

### 2.5. Statistical Analysis

Statistical analyses were performed using IBM SPSS Statistics for Windows, version 26.0 (IBM Corp., Armonk, NY, USA). Continuous variables were assessed for normality using the Shapiro–Wilk test and visual inspection of histograms. Continuous variables are presented as mean ± standard deviation (SD) or median with interquartile range (IQR), whereas categorical variables are presented as frequencies and percentages.

Changes in NRS and NDI scores over time were analyzed using the Friedman test, followed by post hoc Wilcoxon signed-rank tests with Bonferroni correction for pairwise comparisons. Changes in GPE and Likert scale scores were evaluated using the marginal homogeneity test, whereas changes in MPR rates were assessed using Cochran’s Q test. Post hoc pairwise comparisons for MPR were performed using the McNemar test with Bonferroni adjustment. The proportion of patients achieving clinically meaningful NDI improvement (≥30% reduction from baseline) was likewise evaluated using Cochran’s Q test, followed by pairwise McNemar tests with Bonferroni adjustment.

Given the limited number of 6-month MPR events, multivariable binary logistic regression was performed as an exploratory analysis to examine factors independently associated with MPR at 6 months. Variables considered clinically relevant were entered into the multivariable model using the enter method; estimates were interpreted cautiously because of the limited number of outcome events relative to the number of variables included. Odds ratios (ORs) with 95% confidence intervals (CIs) were calculated. Model performance was assessed using the Hosmer–Lemeshow goodness-of-fit test, the Omnibus test of model coefficients, Nagelkerke’s R^2^, and classification accuracy.

Receiver operating characteristic (ROC) curve analysis was performed to evaluate the ability of baseline NRS and NDI scores to predict MPR at 6 months. The AUC with 95% CIs was calculated. A two-sided *p* value < 0.05 was considered statistically significant. For repeated categorical and ordinal outcomes, the reported proportions and their within-patient changes across follow-up time points directly convey the magnitude of effect; standardized effect size indices were not additionally calculated for these paired comparisons. This study is reported in accordance with the Strengthening the Reporting of Observational Studies in Epidemiology (STROBE) guideline for cohort studies (Supplementary Table S1).

## 3. Results

Of the patients who underwent fluoroscopy-guided cervical medial branch PRF for chronic cervical facet joint pain between 2020 and 2025, 75 met the predefined inclusion criteria and were included in the final analysis.

Table 1 summarizes the baseline demographic and clinical characteristics of the 75 patients included in the study. The mean age was 61.7 ± 11.3 years, and the sex distribution was nearly equal (50.7% female and 49.3% male). The procedure was performed more frequently on the right side (60.0%) than on the left (40.0%). The most common treatment level was C3–4, C4–5, and C5–6 (58.7%), followed by C4–5, C5–6, and C6–7 (41.3%). The most prevalent comorbidities were hypertension (61.3%) and fibromyalgia syndrome (56.0%), followed by rheumatoid arthritis (40.0%), diabetes mellitus (38.7%), and thyroid disease (38.7%).

**Figure 2:**
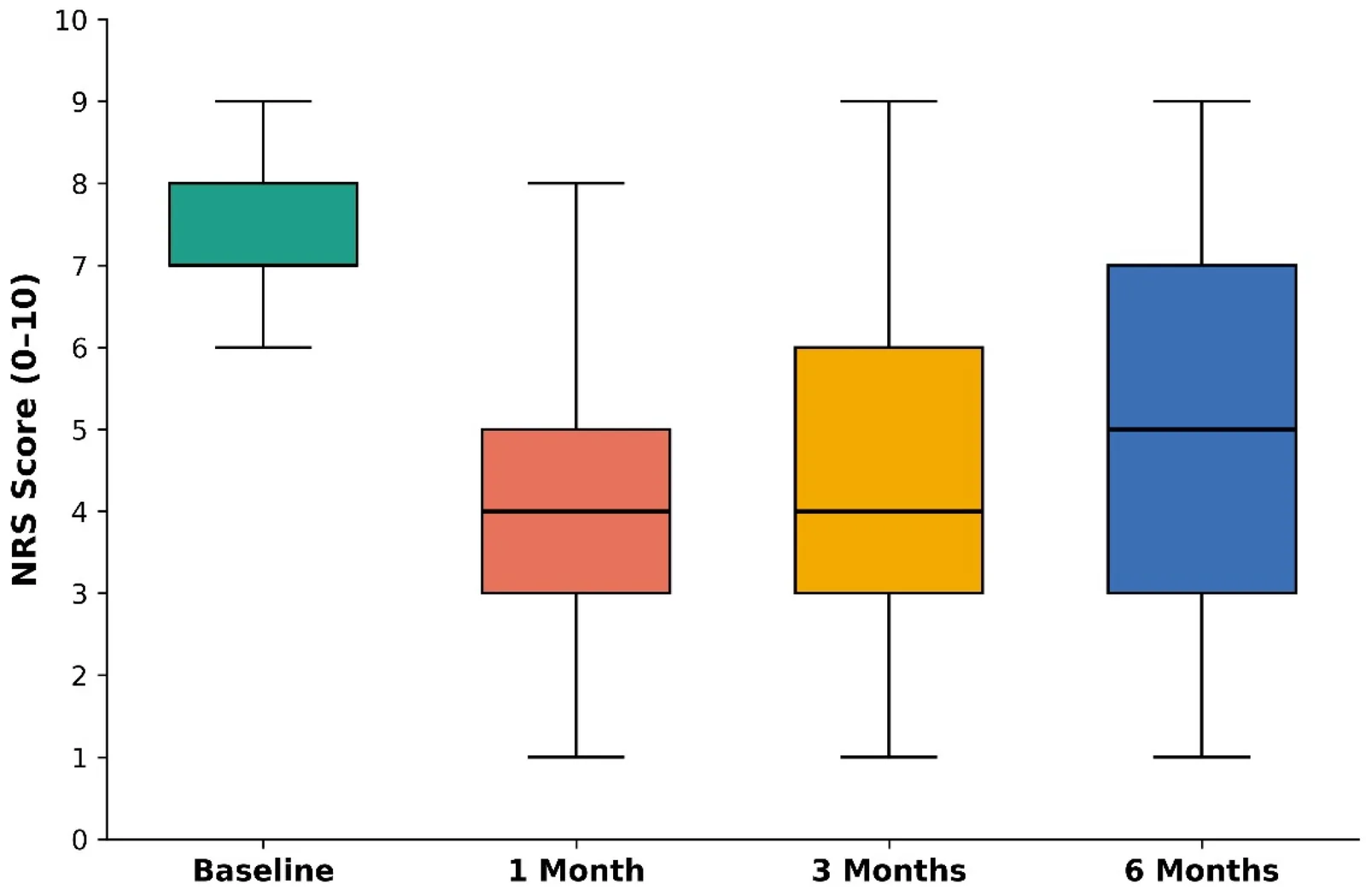
Distribution of Numeric Rating Scale (NRS) scores during the 6-month follow-up. Box plots show the median, the interquartile range (IQR), and the minimum and maximum values.

**Figure 3:**
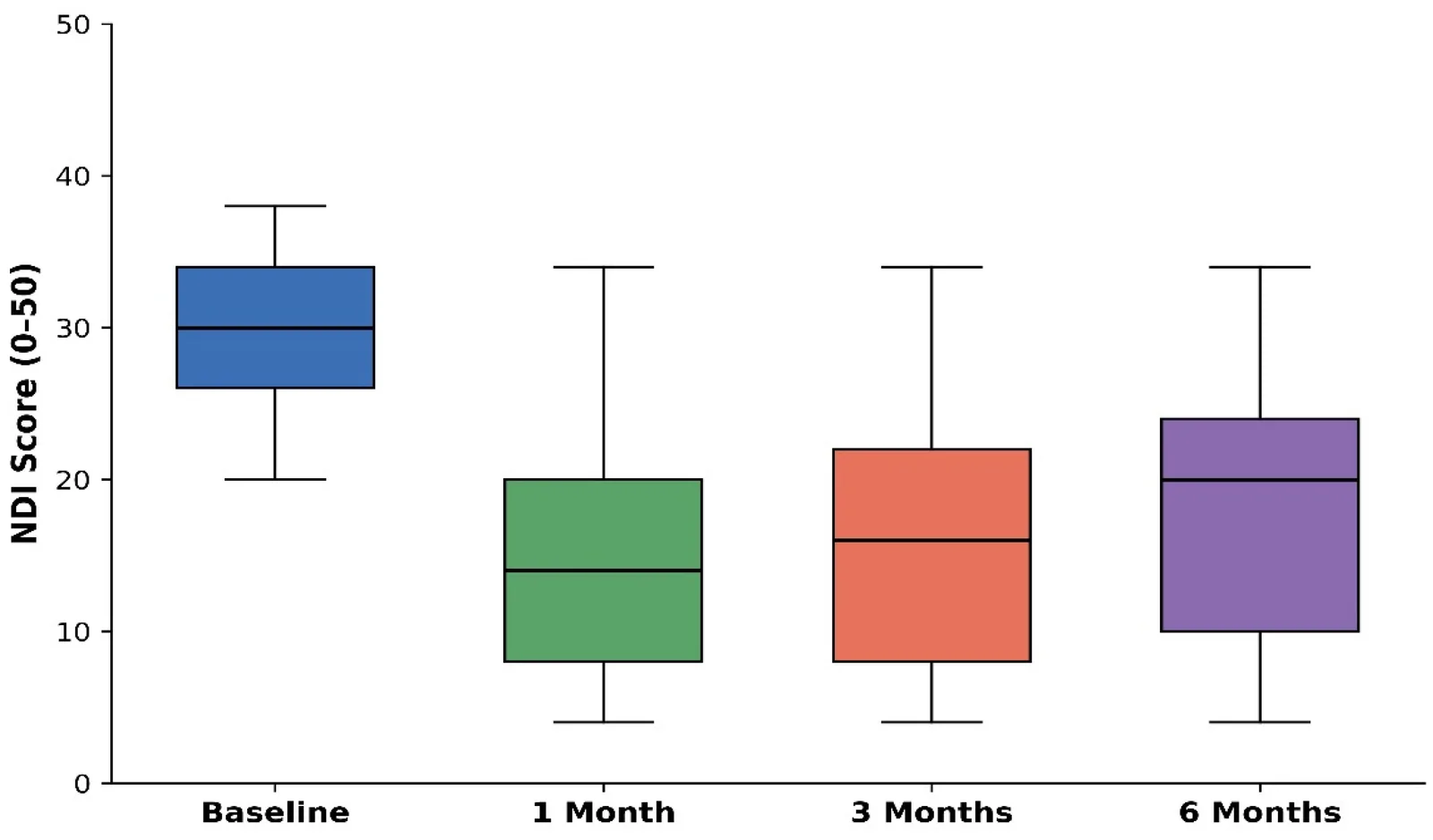
Distribution of Neck Disability Index (NDI) scores during the 6-month follow-up. Box plots show the median, the interquartile range (IQR), and the minimum and maximum values.

Table 2 demonstrates significant improvements in pain intensity, disability, and patient-reported outcomes over the 6-month follow-up period. Median NRS scores decreased from 7.0 (IQR, 1.0) at baseline to 4.0 (IQR, 2.0) at 1 month and 4.0 (IQR, 3.0) at 3 months, and remained significantly lower than baseline at 5.0 (IQR, 4.0) after 6 months (overall *p* < 0.001). Similarly, median NDI scores improved significantly from 30.0 (IQR, 8.0) at baseline to 14.0 (IQR, 12.0) at 1 month, 16.0 (IQR, 14.0) at 3 months, and 20.0 (IQR, 14.0) at 6 months (overall *p* < 0.001). NRS and NDI scores during follow-up are demonstrated in Figure 2 and Figure 3, respectively. Both GPE scores and the 3-point Likert-scale-rated change in analgesic medication use changed significantly across follow-up compared with the 1-month assessment (*p* < 0.001). Patient-reported global improvement declined over time, although a subset of patients continued to report substantial improvement at 6 months, whereas an increasing proportion of patients reported no reduction in analgesic medication use over time, a pattern paralleling the decline in NRS-defined MPR described below.

**Figure 4:**
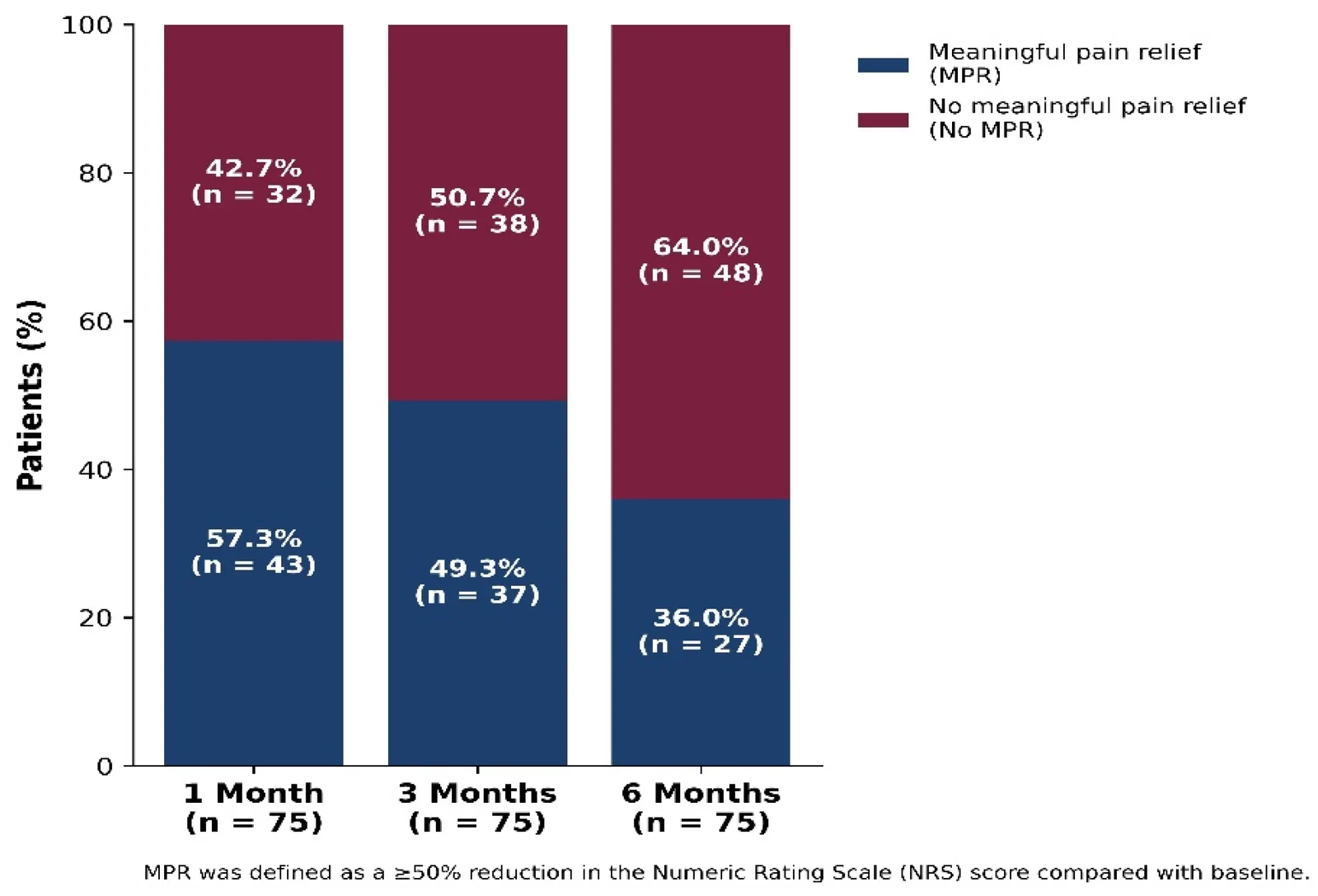
Proportion of patients achieving meaningful pain relief (MPR) during the 6-month follow-up. MPR was defined as a ≥50% reduction in the Numeric Rating Scale (NRS) score compared with baseline.

Meaningful pain relief rates were significantly different over time (Cochran’s Q *p* < 0.001). At 1 month, 43 patients (57.3%) achieved meaningful pain relief (MPR), defined as ≥50% decrease in NRS scores (Table 3). At 3 months, the proportion of patients achieving MPR decreased to 37 (49.3%); however, this reduction was not statistically significant compared with the 1-month follow-up (*p* = 0.199). At 6 months, the MPR ratio further declined to 27 patients (36.0%), representing a significant reduction compared with the 1-month follow-up (*p* < 0.001). The MPR rate at 6 months was also significantly lower than at 3 months (*p* = 0.007), confirming a continued decline in treatment response between these two follow-up time points. Expressed as an absolute risk difference, the MPR rate declined by 21.3 percentage points between 1 and 6 months (57.3% to 36.0%), providing a directly interpretable measure of the magnitude of attenuation in treatment response over time. Figure 4 shows the MPR ratio for the patients during follow-up.

**Figure 5:**
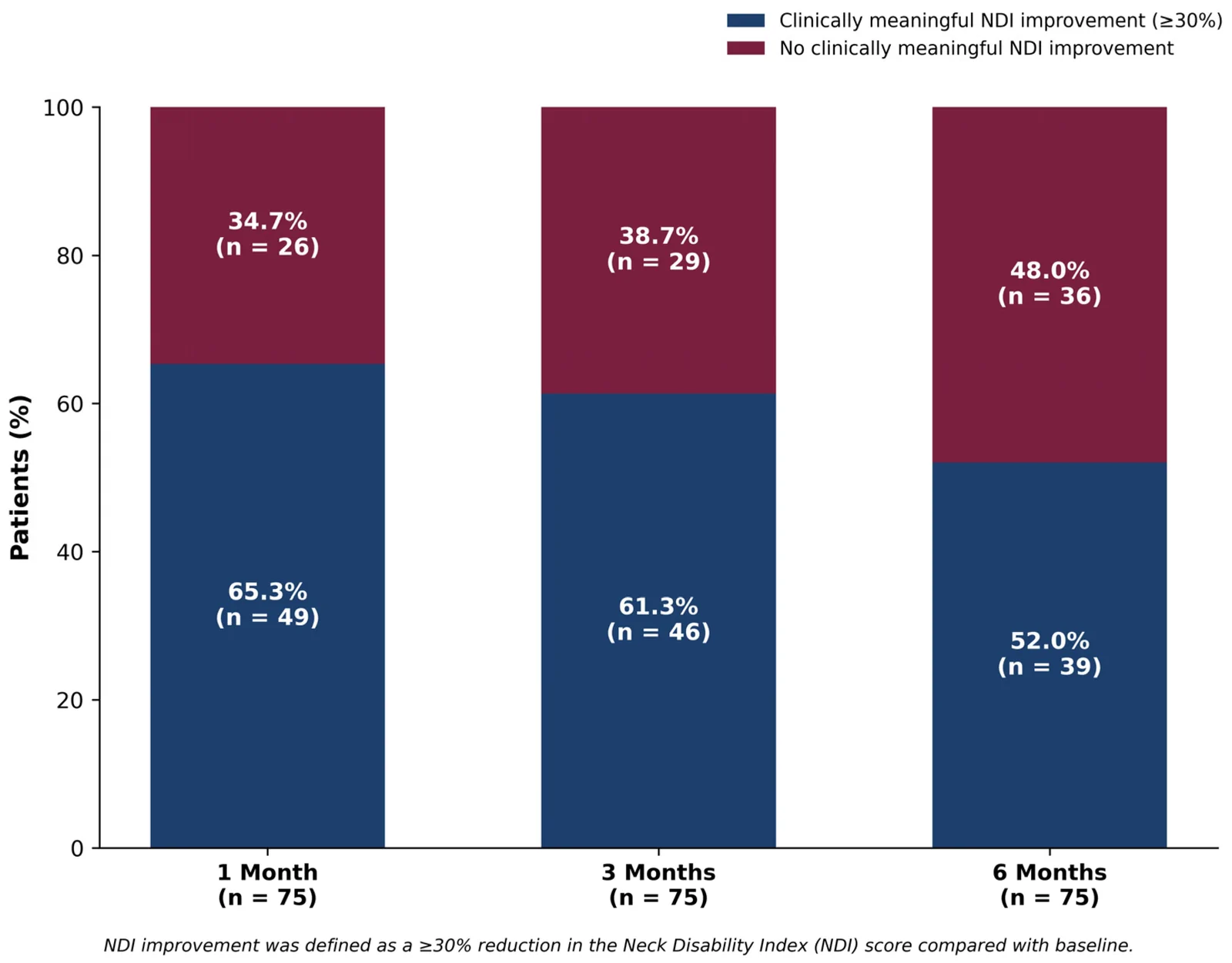
Proportion of patients achieving clinically meaningful improvement in the Neck Disability Index (NDI) during the 6-month follow-up. Clinically meaningful improvement was defined as a ≥30% reduction in NDI score compared with baseline.

The proportion of patients achieving a clinically meaningful improvement in NDI (defined as a ≥30% reduction from baseline) was 65.3% at month 1, 61.3% at month 3, and 52.0% at month 6 (Figure 5). The overall distribution of clinically meaningful NDI improvement differed significantly during follow-up (Cochran’s Q test, *p* < 0.001). In pairwise comparisons, the proportion of patients achieving meaningful improvement did not differ significantly between months 1 and 3 (*p* = 0.736), whereas it was significantly lower at month 6 than at month 1 (*p* < 0.001) and month 3 (*p* = 0.020) after Bonferroni correction (Table 4).

An exploratory multivariable logistic regression analysis was performed using clinically relevant variables, including age, sex, fibromyalgia, and baseline NRS. The overall model was statistically significant (Omnibus test, *p* = 0.046), showed no evidence of poor calibration (Hosmer–Lemeshow test, *p* = 0.454), had an overall classification accuracy of 65.3%, and yielded a Nagelkerke R^2^ of 0.166. Within this exploratory model, fibromyalgia was the only variable independently associated with 6-month MPR and was associated with lower odds of achieving MPR (OR = 0.25, 95% CI 0.088–0.709, *p* = 0.009). Age, sex, and baseline NRS were not significantly associated with the outcome (Table 5). The 6-month MPR rate differed significantly according to fibromyalgia status: among patients with fibromyalgia (*n* = 42), 9 (21.4%) achieved MPR, compared with 18 of 33 (54.5%) patients without fibromyalgia (χ^2^ = 8.797, *p* = 0.003). A similar, non-significant trend was observed for rheumatoid arthritis: 7 of 30 (23.3%) patients with rheumatoid arthritis achieved MPR, compared with 20 of 45 (44.4%) patients without rheumatoid arthritis (χ^2^ = 3.482, *p* = 0.062).

Table 6 presents the ROC analysis evaluating the ability of baseline pain intensity and disability to predict MPR at 6 months. Neither baseline NRS nor baseline NDI demonstrated significant discriminatory performance. Baseline NRS yielded an AUC of 0.584 (95% CI, 0.446–0.723; *p* = 0.227), while baseline NDI showed an AUC of 0.558 (95% CI, 0.418–0.698; *p* = 0.408), indicating poor discrimination for 6-month MPR.

No serious acute periprocedural adverse events were documented during the 2 h observation period.

## 4. Discussion

The combined intervention of fluoroscopy-guided cervical medial branch PRF with post-procedural local anesthetic and corticosteroid administration was associated with significant reductions in both pain intensity and neck-related disability, with the greatest benefit observed during the early follow-up period and partial attenuation by 6 months. Median NRS scores decreased from 7.0 at baseline to 4.0 at 1 and 3 months before rising to 5.0 at 6 months, while NDI scores followed a parallel trajectory, improving from 30.0 to 14.0 at 1 month before drifting upward to 20.0 by 6 months (both overall *p* < 0.001). This pattern of rapid early improvement followed by partial attenuation, rather than sustained maximal benefit, foreshadows the parallel decline in the proportion of patients achieving MPR discussed below.

One plausible explanation for this temporal pattern relates to the non-destructive, neuromodulatory mechanism of PRF outlined in the Introduction; however, because this study was retrospective, uncontrolled, and involved co-administration of corticosteroid and local anesthetic at each session, this mechanistic interpretation could not be directly tested against the present data and remains hypothesis-generating. Unlike conventional (thermal) radiofrequency ablation, which produces a coagulative lesion and more durable denervation, PRF modulates nociceptive signaling without inducing frank axonal destruction, and its clinical benefit may therefore diminish over time as neuromodulatory effects subside. This expectation is supported by direct comparative data from lumbar facet joint pain: in a three-arm trial comparing local anesthetic, conventional radiofrequency, and PRF, pain relief was maintained in the conventional radiofrequency group at 6 and 12 months but had already dissipated in the PRF group over the same interval, with the highest rate of analgesic discontinuation and patient satisfaction also observed in the conventional radiofrequency group [15]. A recent single-center comparison of cervical medial branch PRF and conventional radiofrequency similarly found no between-group difference at 1 and 3 months, but significantly better pain, disability, and satisfaction outcomes with conventional radiofrequency at 6 and 12 months [16]. Long-term cohorts of conventional cervical radiofrequency neurotomy selected by rigorous comparative diagnostic blocks have reported relief lasting approximately 17–20 months after the initial procedure, with a median cumulative relief of 20–26 months when repeat treatment was considered and roughly 60% of patients still benefiting at follow-up [17]—a durability substantially longer than that observed for PRF in the present cohort. Taken together, these findings are consistent with the possibility that the attenuation of benefit observed by 6 months may relate, at least in part, to the predominantly neuromodulatory rather than neuroablative nature of PRF, rather than to a failure of technique or patient selection.

The proportion of patients achieving MPR in the present study—57.3% at 1 month, 49.3% at 3 months, and 36.0% at 6 months—is broadly consistent with prior reports of cervical medial branch PRF. Chang and Yang reported that 52.4% of patients achieved clinically meaningful relief at 3 months [9], closely matching our 3-month rate. At 6 months, our responder rate of 36.0% approximates the 35.2% success rate reported in a recent cohort using the same outcome definition and a comparable follow-up schedule [10], while in a larger cohort, Yen et al. reported a good outcome, defined as ≥50% pain reduction, in 71.3% of patients who underwent PRF (112/157) and identified sagittal alignment parameters, including the C2–C7 Cobb angle, as independent predictors of outcome [8]. More recently, Aktan and Yanık evaluated 177 patients undergoing cervical medial branch PRF under fluoroscopic or ultrasound guidance and reported comparable clinical outcomes between imaging modalities across NRS, NDI, and analgesic-use measures, with follow-up extending to 6 months [18]. The convergence of our 3- and 6-month responder rates with those of comparably designed studies lends external validity to our findings and reinforces the broader evidence of clinical improvement following cervical medial branch PRF, while our serial responder analysis additionally demonstrates attenuation of the observed response over time.

Importantly, the improvement in pain intensity observed in this cohort was accompanied by a parallel improvement in neck-related disability, indicating that the observed clinical benefit extended beyond the pain domain to functional status. This concordance between pain and disability outcomes has been reported in other cervical PRF and radiofrequency cohorts using the NDI, including the comparative fluoroscopy- versus ultrasound-guided cohort described above [10,16,18]. Together, these observations support the clinical relevance of the pain reduction observed here rather than reflecting an isolated change in a single subjective measure.

A clinically distinctive finding of this exploratory analysis was that fibromyalgia, rather than age, sex, or baseline pain intensity, was independently associated with lower odds of achieving MPR at 6 months; however, this association should be interpreted cautiously given the limited number of 6-month MPR events and the resulting potential for model instability. This finding is biologically plausible. Fibromyalgia is considered a prototypical central sensitization syndrome, characterized by amplified central pain processing, hyperalgesia, expanded receptive fields, and impaired descending inhibitory control [19]. PRF directed at a peripheral target—the medial branch nerve—may therefore be less effective when a substantial component of the pain experience is maintained by central mechanisms. Direct evidence examining centralized pain in patients undergoing medial branch radiofrequency treatment remains limited. Morffi et al. prospectively evaluated patients undergoing medial branch RFA using the 2011 Fibromyalgia Survey Criteria and found that patients with a centralized pain phenotype showed a trend toward less improvement in overall body pain, although this difference was not statistically significant; importantly, centralized pain status was also not significantly associated with improvement in site-specific spinal pain at 3 months [20]. In contrast, clinically diagnosed fibromyalgia in the present cohort was associated with a substantially lower likelihood of achieving MPR at 6 months in the exploratory multivariable model. Although differences in treatment modality, outcome definition, and follow-up preclude direct comparison, our findings suggest that fibromyalgia or centralized pain characteristics warrant further investigation as potential determinants of the durability of medial branch radiofrequency outcomes.

This interpretation is further supported by a large cohort of patients undergoing surgical, interventional, and injection-based treatments for chronic spinal pain, in which higher Central Sensitization Inventory scores were independently associated with markedly reduced treatment satisfaction (OR = 0.09), while patients with CSI scores ≥40 were also less likely to achieve minimum clinically important improvement in both the Oswestry Disability Index and Neck Disability Index [21]. A similarly directed association between preoperative central sensitization and inferior outcomes has been reported across orthopedic surgical populations, including hip and knee arthroplasty [22], suggesting that this relationship is not specific to spinal interventions but may reflect a broader phenomenon relevant to procedural pain management.

The association observed with fibromyalgia contrasts with factors reported in other cervical radiofrequency cohorts, which have more commonly emphasized structural, procedural, or symptom-based characteristics. In a multicenter analysis of cervical facet radiofrequency denervation, paraspinal tenderness was the only clinical variable associated with success, while opioid use, pain radiating to the head, and pain exacerbated by extension or rotation were associated with failure [23]. A recent cohort using an identical MPR definition to the present study identified paraspinal tenderness, shorter pain duration, and lower baseline pain and disability as independent predictors of success [10]. Neither of these studies examined fibromyalgia or other central sensitization-related comorbidities as candidate predictors, and our findings suggest that such comorbidities represent an underexplored dimension warranting further investigation; prospective validation is required before these factors are used to guide patient selection.

Consistent with the heterogeneity of predictor profiles described above, baseline NRS and NDI demonstrated poor discriminatory ability for 6-month MPR (AUC 0.584 and 0.558, respectively), while baseline NRS was not significantly associated with MPR in the multivariable model. This finding suggests that baseline pain or disability severity alone may have limited value in identifying patients likely to experience benefit from the combined intervention and supports further investigation of comorbidity and pain-phenotype assessment in prospective studies.

Beyond the strict MPR threshold, the proportion of patients reporting a favorable global impression (GPE score ≥6) and the proportion reporting a reduction in analgesic medication use followed a similar downward trajectory to MPR itself, declining from 62.7% and 70.7%, respectively, at 1 month to 30.7% and 37.3% at 6 months. This concordance across three complementary clinical outcome measures—pain-based responder status, global impression of change, and medication-use pattern—suggests that the waning of treatment benefit observed in this cohort reflects a genuine decline in overall clinical benefit, rather than an artifact specific to the NRS-based MPR threshold. No serious acute periprocedural adverse events were documented during the 2 h observation period, consistent with the limited acute safety ascertainment available in this retrospective cohort.

These findings may have practical implications for clinical decision-making. The observed attenuation of treatment response over time suggests that patients undergoing cervical medial branch PRF should be counseled regarding the possibility that benefit associated with the combined intervention may diminish during follow-up. In addition, the association between fibromyalgia and lower odds of 6-month MPR suggests that the presence of fibromyalgia may be relevant when discussing expected treatment durability with patients. More broadly, these findings support consideration of pain phenotype and central sensitization-related comorbidities as part of patient assessment, although their role in treatment selection requires confirmation in prospective studies. Finally, the decline in response observed by 6 months highlights the need for further studies to determine whether repeat PRF, conventional radiofrequency, or other adjunctive strategies may provide additional benefit in patients with waning response.

This study has several limitations. Its retrospective, single-center design without a comparator arm (e.g., conventional radiofrequency or sham) precludes causal inference regarding the relative efficacy of PRF. Patient selection was based on ≥50% temporary pain relief following a diagnostic medial branch block. Although more stringent thresholds have been advocated and may enrich for higher degrees of pain relief after thermal radiofrequency neurotomy, cervical facet literature has used cut-offs ranging from 50% to complete pain relief, and no single threshold has consistently been shown to optimize overall radiofrequency outcomes [24,25]. Accordingly, the ≥50% criterion used in the present study represents a pragmatic selection strategy consistent with contemporary consensus recommendations [25]. The 0.5–1.0 mL local anesthetic volume used for the diagnostic medial branch block may also have permitted spread to adjacent structures, potentially reducing diagnostic specificity and increasing the risk of false-positive block responses. Because PRF was routinely accompanied by co-administration of local anesthetic and corticosteroid at the treated levels, the independent contribution of PRF itself cannot be fully isolated from these adjunct effects. Follow-up was limited to 6 months, precluding assessment of longer-term durability and of whether repeat PRF could restore meaningful relief once the initial effect attenuates. In addition, inclusion was restricted to patients with complete 6-month follow-up data, which may have introduced selection bias by excluding patients lost to follow-up. In patients with bilateral symptoms, only one clinically predominant side was included as the index treated side in the analysis. Contralateral symptoms could nevertheless have influenced patient-reported NRS and NDI scores during follow-up: persistent untreated contralateral pain could attenuate the apparent magnitude of improvement associated with the index treatment, whereas any subsequent contralateral treatment could also affect overall patient-reported scores. Because the course of contralateral symptoms and the timing of any subsequent contralateral procedures were not analyzed as study variables, the magnitude and direction of this potential influence cannot be quantified. Finally, the sample size, while comparable to similar retrospective cohorts, limited the power to detect additional, smaller-magnitude associations. In particular, with only 27 patients achieving the 6-month MPR endpoint and four variables included in the multivariable model, the exploratory regression analysis remains vulnerable to model instability; therefore, the observed association with fibromyalgia should be interpreted cautiously and requires confirmation in larger cohorts. Fibromyalgia diagnoses were based on pre-existing clinical diagnoses recorded in the electronic health record rather than formal, study-specific application of American College of Rheumatology diagnostic criteria, which may introduce diagnostic heterogeneity. Baseline psychological characteristics were not systematically assessed using validated instruments for anxiety, depression, or psychological distress. Because these factors may influence pain intensity and disability and may overlap with centralized pain phenotypes, residual confounding of the observed association between fibromyalgia and 6-month MPR cannot be excluded. Information on analgesic medication use was based on patients’ self-reported overall change in consumption (discontinued, decreased, or unchanged) rather than systematically extracted medication classes, formulations, or doses for each patient. Most patients used topical non-steroidal anti-inflammatory preparations, with a smaller subset using opioid analgesics; however, because exact opioid doses were not consistently documented in the clinical records, standardized metrics such as Morphine Milligram Equivalents (MME) could not be calculated. This represents a limitation of the present study. Outcome assessments were performed by pain specialists within the treating department rather than by independent or blinded assessors external to the clinical team, which may have introduced observer bias, particularly for patient-reported outcomes such as GPE. Some patients had a more remote history of other cervical interventions (e.g., trigger point injections or epidural steroid injections) documented in their clinical records; however, the type, timing, and frequency of such prior interventions were not systematically extracted as a study variable. This is consistent with the inclusion criterion requiring an inadequate response to conservative treatment, but precludes quantitative reporting of prior intervention history in Table 1.

## 5. Conclusions

Fluoroscopy-guided cervical medial branch pulsed radiofrequency, combined with post-procedural corticosteroid and local anesthetic administration, was associated with significant reductions in pain intensity and neck-related disability in patients with chronic cervical facet joint pain, with the greatest benefit associated with the combined intervention observed during the first 3 months after treatment. Although pain and disability outcomes remained significantly improved compared with baseline at 6 months, the proportion of patients achieving meaningful pain relief progressively decreased over time, indicating attenuation of the observed treatment response. In the exploratory multivariable analysis, fibromyalgia was independently associated with a lower likelihood of achieving meaningful pain relief at 6 months, whereas age, sex, and baseline pain intensity were not significantly associated with 6-month MPR. These findings suggest that this combined intervention was associated with clinically meaningful short- to medium-term improvement in appropriately selected patients, while the presence of fibromyalgia may be relevant when counseling patients regarding the durability of treatment response. Prospective controlled studies with larger samples and longer follow-up are warranted to confirm these findings and further define factors associated with sustained response.

## Figures and Tables

**Table 1:** Baseline demographic and clinical characteristics of the study population (*n* = 75).

Age (Mean ± SD)	61.7 ± 11.3
MRI-confirmed facet joint degeneration (*n*/%)	75 (100.0)
Sex (*n*/%)	
Female	38 (50.7)
Male	37 (49.3)
Side of the procedure (*n*/%)	
Right	45 (60.0)
Left	30 (40.0)
Level of treatment (*n*/%)	
C3-4, C4-5, C5-6	44 (58.7)
C4-5, C5-6, C6-7	31 (41.3)
Comorbidities (*n*/%)	
HT	46 (61.3)
DM	29 (38.7)
Thyroid disease	29 (38.7)
Fibromyalgia syndrome	42 (56.0)
Rheumatoid arthritis	30 (40.0)

Data are presented as mean ± standard deviation (SD) or number (percentage). SD: Standard deviation, HT: hypertension; DM: diabetes mellitus.

**Table 2:** Changes in pain intensity and patient-reported outcomes during the follow-up.

Variable		*p*
NRS (median:IQR)		<0.001 ^a^
Baseline	7.0 (1.0)	
Month 1	4.0 (2.0)	<0.001 ^b^
Month 3	4.0 (3.0)	<0.001 ^b^
Month 6	5.0 (4.0)	<0.001 ^b^
NDI (median: IQR)		<0.001 ^a^
Baseline	30.0 (8.0)	
Month 1	14.0 (12.0)	<0.001 ^b^
Month 3	16.0 (14.0)	<0.001 ^b^
Month 6	20.0 (14.0)	<0.001 ^b^
GPE (*n*/%)		
Month 1		
4	12 (16.0)	
5	16 (21.3)	
6	19 (25.3)	
7	28 (37.3)	
Month 3		<0.001 ^c^
4	23 (30.7)	
5	16 (21.3)	
6	15 (20.0)	
7	21 (28.0)	
Month 6		<0.001 ^c^
4	27 (36.0)	
5	25 (33.3)	
6	4 (5.3)	
7	19 (25.3)	
Likert scale (*n*/%)		
Month 1		
0	21 (28.0)	
1	32 (42.7)	
2	22 (29.3)	
Month 3		<0.001 ^c^
0	17 (22.7)	
1	19 (25.3)	
2	39 (52.0)	
Month 6		<0.001 ^c^
0	6 (8.0)	
1	22 (29.3)	
2	47 (62.7)	

Values are presented as median (interquartile range [IQR]) or number (percentage). ^a^ Friedman test; ^b^ Post hoc pairwise comparisons with baseline using the Wilcoxon signed-rank test with Bonferroni correction; ^c^ Marginal homogeneity test comparing follow-up visits with the 1-month assessment. Percentages may not total 100% due to rounding. NRS: Numeric Rating Scale; NDI: Neck Disability Index; GPE: Global Perceived Effect; IQR: interquartile range.

**Table 3:** Changes in the rates of patients achieving meaningful pain relief during follow-up.

Meaningful Pain Relief (*n*/%)		*p* *
Month 1	43 (57.3)	
Month 3	37 (49.3)	0.199
Month 6	27 (36.0)	<0.001
Month 3 vs. Month 6		0.007

* *p*-values indicate pairwise comparisons performed with the McNemar test and Bonferroni correction following Cochran’s Q test. Overall comparison across follow-up time points: Cochran’s Q test, *p* < 0.001.

**Table 4:** Clinically meaningful improvement in the Neck Disability Index over follow-up.

NDI Improvement (≥30% Decrease in Score)		*p* *
Month 1	49 (65.3)	
Month 3	46 (61.3)	0.736
Month 6	39 (52.0)	<0.001
Month 3 vs. Month 6		0.020

* *p*-values indicate pairwise comparisons performed with the McNemar test and Bonferroni correction following Cochran’s Q test. Overall comparison across follow-up time points: Cochran’s Q test, *p* < 0.001.

**Table 5:** Unadjusted and multivariable-adjusted logistic regression analysis examining factors associated with meaningful pain relief at 6 months.

Variable	Unadjusted OR (95% CI)	*p*	Adjusted OR (95% CI)	*p*
Age	1.019 (0.976–1.063)	0.394	1.008 (0.962–1.056)	0.749
Sex (Ref: Female)	1.477 (0.573–3.812)	0.420	1.239 (0.434–3.535)	0.689
Fibromyalgia (Ref: No)	0.227 (0.083–0.622)	**0.004**	0.250 (0.088–0.709)	**0.009**
Baseline NRS	1.302 (0.753–2.252)	0.345	1.256 (0.704–2.239)	0.440
Rheumatoid arthritis (Ref: No)	0.380 (0.136–1.066)	0.066	Not included in multivariable model	—

OR: Odds ratio; CI: Confidence interval; NRS: Numeric Rating Scale. Bold values indicate statistical significance (*p* < 0.05). Rheumatoid arthritis was assessed univariately but not included in the multivariable model due to the limited number of events relative to the number of candidate predictors (events-per-variable constraint).

**Table 6:** Receiver operating characteristic (ROC) analysis of baseline NRS and NDI scores for predicting meaningful pain relief at 6 months.

Predictor	AUC	95% CI	*p* Value
Baseline NRS	0.584	0.446–0.723	0.227
Baseline NDI	0.558	0.418–0.698	0.408

ROC: Receiver operating characteristic; AUC: Area under the curve; CI: Confidence interval; NRS: Numeric Rating Scale; NDI: Neck Disability Index.

## Data Availability

The datasets generated and analyzed during the current study are not publicly available due to ethical and privacy restrictions but are available from the corresponding author on reasonable request.

## Supplementary Materials

The following supporting information can be downloaded at https://www.mdpi.com/article/10.3390/medicina62091770/s1. Table S1: STROBE Statement—checklist of items for reports of cohort studies; Figure S1: Participant flow diagram.

## Abbreviations

The following abbreviations are used in this manuscript:

AP	Anteroposterior
AUC	Area under the curve
CI	Confidence interval
CSI	Central Sensitization Inventory
DM	Diabetes mellitus
GPE	Global Perceived Effect
HT	Hypertension
IQR	Interquartile range
MBB	Medial branch block
MCID	Minimum clinically important difference
MPR	Meaningful pain relief
NDI	Neck Disability Index
NRS	Numeric Rating Scale
OR	Odds ratio
PRF	Pulsed radiofrequency
RF	Radiofrequency
RFA	Radiofrequency ablation
ROC	Receiver operating characteristic
