# Supplementary material for: Clinical Outcomes and Factors Associated with Response to Fluoroscopy-Guided Cervical Medial Branch Pulsed Radiofrequency in Chronic Cervical Facet Joint Pain: A Retrospective Cohort Study
Source: Medicina (Kaunas). 2026 Sep 15;62(9):1770. doi: 10.3390/medicina62091770 (PMC13609532; doi:10.3390/medicina62091770)
Supplement: Supplementary file 1 [file medicina-62-01770-s001.zip › medicina-4543477-Figure S1 .pdf]

Supplementary Figure S1. Participant flow diagram

Patients who underwent fluoroscopy-guided cervical medial branch PRF
for chronic cervical facet joint pain between 2020 and 2025
(n = 103)

↓

Excluded (n = 28)
Missing 6-month follow-up data (n = 13)
Predefined exclusion criteria (n = 5), most commonly radicular pain attributable to disc herniation or active inflammatory rheumatologic disease requiring systemic corticosteroid treatment
Another invasive cervical procedure in close temporal proximity to PRF (n = 10): cervical epidural steroid injection (n = 3); trigger point injections (n = 7)

↓

Patients meeting all inclusion criteria
and included in the final analysis
(n = 75)

↓

Complete outcome data at baseline and at 1, 3, and 6 months
NRS, NDI, GPE, and analgesic medication-use assessments
(n = 75)

Abbreviations: PRF, pulsed radiofrequency; NRS, Numeric Rating Scale; NDI, Neck Disability Index; GPE, Global Perceived Effect.
