# Supplementary material for: Clinical Outcomes and Factors Associated with Response to Fluoroscopy-Guided Cervical Medial Branch Pulsed Radiofrequency in Chronic Cervical Facet Joint Pain: A Retrospective Cohort Study
Source: Medicina (Kaunas). 2026 Sep 15;62(9):1770. doi: 10.3390/medicina62091770 (PMC13609532; doi:10.3390/medicina62091770)
Supplement: Supplementary file 1 [file medicina-62-01770-s001.zip › medicina-4543477-Table S1.pdf]

Supplementary Table S1. STROBE Statement — Checklist of Items for Reports of Cohort Studies

Manuscript: Clinical Outcomes and Factors Associated with Response to Fluoroscopy-Guided Cervical Medial Branch Pulsed Radiofrequency in Chronic Cervical Facet Joint Pain: A Retrospective Cohort Study

Item	Section/Topic	STROBE Recommendation	Reported on Page/Section
1a	Title and abstract	(a) Indicate the study's design with a commonly used term in the title or the abstract	Indicated in the title: "...A Retrospective Cohort Study"
1b		(b) Provide in the abstract an informative and balanced summary of what was done and what was found	Structured abstract (Background and Objectives, Materials and Methods, Results, Conclusion)
2	Background/rationale	Explain the scientific background and rationale for the investigation being reported	Introduction, paragraphs 1–5
3	Objectives	State specific objectives, including any prespecified hypotheses	Introduction, final paragraph
4	Study design	Present key elements of study design early in the paper	Section 2.1 (Study Design and Setting)
5	Setting	Describe the setting, locations, and relevant dates, including periods of recruitment, exposure, follow-up, and data collection	Section 2.1; treatment period 2020–2025 stated
6a	Participants	(a) Give the eligibility criteria, and the sources and methods of selection of participants	Section 2.2, full inclusion/exclusion criteria
6b		(b) For matched studies, give matching criteria and number of exposed and unexposed	Not applicable — study is not matched
7	Variables	Clearly define all outcomes, exposures, predictors, potential confounders, and effect modifiers	Section 2.4 (Outcome Measures); NRS, NDI, GPE, Likert, and MPR defined
8	Data sources/measurement	For each variable of interest, give sources of data and details of methods of assessment	Section 2.1 (electronic hospital records) and Section 2.4
9	Bias	Describe any efforts to address potential sources of bias	Section 2.2 — inclusion restricted to patients with complete outcome data at all follow-up time points, to minimize selection bias
10	Study size	Explain how the study size was arrived at	Section 2.2 — no a priori sample size calculation performed; all eligible patients during the study period were included
11	Quantitative variables	Explain how quantitative variables were handled in the analyses; describe grouping and why	Section 2.4 — MPR defined a priori as ≥50% NRS reduction; NDI severity categories defined
12a	Statistical methods	(a) Describe all statistical methods, including those used to control for confounding	Section 2.5
12b		(b) Describe any methods used to examine subgroups and interactions	Not applicable — no subgroup/interaction analyses were performed
12c		(c) Explain how missing data were addressed	Section 2.2 — patients with missing 6-month follow-up data were excluded a priori
12d		(d) If applicable, explain how loss to follow-up was addressed	Section 2.2 exclusion criteria; addressed further as a limitation in the Discussion
12e		(e) Describe any sensitivity analyses	Not performed — noted as a direction for future research
13a	Participants	(a) Report numbers of individuals at each stage of the study	Final analytic cohort (n = 75) reported in Results; of 103 patients screened, participant flow with reasons for exclusion at each stage is detailed in Supplementary Figure S1
13b		(b) Give reasons for non-participation at each stage	Exclusion criteria listed in Section 2.2; stepwise counts provided in Supplementary Figure S1
13c		(c) Consider use of a flow diagram	Flow diagram provided (Supplementary Figure S1)
14a	Descriptive data	(a) Give characteristics of study participants and information on exposures and potential confounders	Table 1
14b		(b) Indicate number of participants with missing data for each variable of interest	Not applicable — only patients with complete data were included (Section 2.2)

Item	Section/Topic	STROBE Recommendation	Reported on Page/Section
14c		(c) For cohort studies, summarize follow-up time	Fixed follow-up schedule (1, 3, and 6 months) stated in Section 2.4
15	Outcome data	Report numbers of outcome events or summary measures over time	Tables 2 and 3; Figures 2–5
16a	Main results	(a) Give unadjusted estimates and, if applicable, confounder-adjusted estimates and their precision (e.g., 95% CI)	Table 5 (adjusted odds ratios with 95% CI)
16b		(b) Report category boundaries when continuous variables were categorized	MPR threshold (≥50% NRS reduction) defined in Section 2.4
16c		(c) If relevant, translate relative risk into absolute risk	Not applicable
17	Other analyses	Report other analyses done — e.g., subgroup, interactions, sensitivity analyses	Table 6 (ROC analysis of baseline NRS and NDI)
18	Key results	Summarize key results with reference to study objectives	Discussion, first paragraph
19	Limitations	Discuss limitations, taking into account sources of potential bias or imprecision; discuss direction and magnitude of any potential bias	Discussion, Limitations paragraph
20	Interpretation	Give a cautious overall interpretation considering objectives, limitations, multiplicity of analyses, results from similar studies, and other relevant evidence	Discussion, throughout
21	Generalisability	Discuss the generalisability (external validity) of the study results	Discussion, Limitations paragraph (single-center design)
22	Funding	Give the source of funding and the role of the funders for the present study and, if applicable, for the original study on which the present article is based	Funding statement (Back Matter)

Note: An Explanation and Elaboration article discusses each checklist item and gives methodological background and published examples of transparent reporting. The STROBE checklist is best used in conjunction with this article, freely available at www.strobe-statement.org.
